# Effect of Anodic Aluminium Oxide Structure on the Electroless Ni-P Distribution into Nanopores

**DOI:** 10.3390/ma18163797

**Published:** 2025-08-13

**Authors:** Boriana Tzaneva, Olena Okhay, Vesselina Milusheva, Stela Atanasova-Vladimirova, João Ventura, Alexander Tkach

**Affiliations:** 1 Department of Chemistry, Technical University of Sofia, 1000 Sofia, Bulgaria; 2 TEMA-Centre for Mechanical Technology and Automation, Department of Mechanical Engineering, University of Aveiro, 3810-193 Aveiro, Portugal; 3 Institute of Physical Chemistry “Rostislav Kaishev”, Bulgarian Academy of Sciences, 1113 Sofia, Bulgaria; 4 IFIMUP—Institute of Physics for Advanced Materials, Nanotechnology and Photonics, Departamento de Fisica e Astronomia, Faculdade de Ciências, Universidade do Porto, Rua do Campo Alegre s/n, 4169-007 Porto, Portugal; 5 CICECO–Aveiro Institute of Materials, Department of Materials and Ceramic Engineering, University of Aveiro, 3810-193 Aveiro, Portugal

**Keywords:** anodic aluminium oxide, electroless deposition, nickel–phosphorus alloy, nanopores

## Abstract

The anodization of aluminium/aluminium alloys is widely used to produce anodic nanoporous networks for metal layered structures, with applications in energy harvesting technologies and sensor systems. Anodic aluminium oxide (AAO) with thickness of ~10 μm and average pore diameter of 13, 33, and 95 nm is prepared by tuning acids and voltages, being further used for electroless nickel deposition, performed for 10 min using conventional electrolyte with sodium hypophosphite reductor and pH 4.5. The formation of Ni nanotubes or nanorods is found to be strongly dependent on AAO pore size. Ni is detected in the whole pore depth and found to form 5–7 μm long continuous tube-like structures only in AAO with pore diameter of 95 nm, being kept just on the AAO top for smaller pore diameters. Nickel distribution in pores along cross-section of AAO is studied as well revealing continuously decreasing ratio to phosphorus amount. The magnetic properties of the resulting Ni 3D structure of a flat conductive layer and nanotubes perpendicular to it do not show significant differences in parallelly and perpendicularly oriented magnetic fields. These observations are discussed considering possible formation mechanisms for an electroless deposited Ni layer on AAO with different structures.

## 1. Introduction

Wet metal deposition methods such as electroless plating and electrodeposition are widely used for the surface modification of materials [1]. Electroless deposition (ELD) is a coating method performed onto substrates using an autocatalytic process without the use of external electrical and/or other additional fields in contrast to electrodeposition [2]. Moreover, ELD is a simple, fast, and low-cost process that is based on a chemical reaction and can provide a uniform coating layer on irregularly shaped workpieces or the entire surface of an item. In particular, electroless nickel depositions are widely used in practice for the preparation of decorative and functional layers on dielectric surfaces such as various ceramics [3], plastics [4], or fabrics [5], etc. The electroless deposition of Ni (Ni-ELD) on different metal supports including aluminium alloys has been widely studied to increase wear and corrosion resistance [6,7]. The electrolyte solutions the most widely used for Ni-ELD are based on NiSO_4_ as a source of nickel ions and various reducing agents, including NaH_2_PO_2_, NaBH_4_, (CH_3_)_2_NHBH_3_, and N_2_H_4_ [8,9,10,11]. Ni-ELD coatings contain varying amounts and elements of the reducing agent such as P and B, depending on the electrolyte composition and pH. For example, when using an acidic hypophosphite electrolyte, lowering the pH from 6.0 to 3.5 leads to a decrease in the deposition rate by more than 4 times, and the phosphorus (P) content in the Ni-P deposit increases from 9 to 13 wt.% [12]. Adjusting the P content in the electroless Ni-P coating is very important, as it determines internal the stress in deposits, their structure, and their magnetic, mechanical, and electrical properties [12]. Increasing the P content from 1 to 12% results in an increase in hardness, the modulus of elasticity, and electrical resistivity. The internal stress in the layer besides being strongly dependent on the substrate undergoes a transition from tensile to compressive stress with increasing P content [12]. Additionally, the presence of P from sodium hypophosphite in an Ni-P deposit is very important since the correct ratio of Ni–P can lead to high corrosion resistance [13,14,15]. The anodic dissolution of metal can be the suppressed because of a P-rich amorphous phase on the surface without boundaries between grains or defects that support corrosion [15,16].

Electroless metal deposition on an anodic aluminium oxide (AAO) template is a promising method in Micro-Electro-Mechanical System technologies and anodic alumina hydrogen permselective composite membranes [17,18], sensors, and catalysts [19] because it allows the building of functional layers and conductive additive patterning on dielectric oxide material. Furthermore, nickel nanowire/nanotube/nanorod arrays obtained using AAO templates can be used to fabricate electrode materials for energy storage devices such as supercapacitors [20,21,22,23,24] and batteries [25,26,27]. Using photolithography, metal layers can perform various functions: electrically and thermally conductive, ferromagnetic, optical, catalytic, etc. At the same time, it was reported that AAO can be used as a scaffold for the nucleation and growth of metal nanoparticles [28,29,30]. However, works reporting the electroless metallization of AAO on an aluminium substrate are rare [17,31,32]. The main reason for this is the high aggressiveness of the activation solutions to both the oxide and the aluminium. Indeed, the choice of the solutions for pretreatment process and for the electroless deposition of anodic oxide layer must be made with caution, since the aluminium oxide obtained by anodization has an amorphous structure. For this reason, AAO has significantly lower chemical resistance in alkaline or strongly acidic environments (especially containing chloride ions) compared to high-temperature modifications of the oxide such as α-Al_2_O_3_, used for ceramics. Therefore, during further processing of AAO on aluminium, its chemical resistance in each specific solution must be considered in order to prevent its local disruption. From this point of view, an Ni-ELD acidic bath with pH range of 4–6 and the reducing agent NaH_2_PO_2_ is more stable for AAO than alkaline baths with a borohydride-reducing agent [1,33]. The resulting Ni-P layer can be used alone as a magnetic shield or as a sublayer on the AAO insulator for subsequent electrodeposition of thick conductive copper layers or other functional coatings.

Nanostructured AAO have some specific features such as high aspect ratio of the pores, hydrophilicity, charge of the pore walls, and high adsorption capacity [34,35,36,37]. These characteristic properties are determined primarily by the used production technology (electrical parameters, temperature, and nature of the electrolyte during anodizing) and by the subsequent surface treatments. Therefore, a strong influence of the morphology, composition, and properties of AAO layers on the penetration depth and uniformity of electroless metal deposition in the pores can be expected. Moreover, the penetration of metal from the electrolyte into the pores of AAO can strongly depend on the pore size or thickness of AAO, as well as on the ratio between components, e.g., nickel and phosphorus. Also, different combinations/ratio between components can lead to the formation of different compounds and/or different nanomaterials with different shapes (nanowires, nanotubes, nanorods, etc.). Therefore, the choice of the pore diameter of the oxide layer can be crucial for obtaining different composite structures: from a surface-localised smooth metal layer to conformal coating of the nanopore walls to the formation of vertical nanotubes with a huge surface area.

The aim of this study is to determine the influence of the diameter of the nanopores of an AAO template on the penetration depth of a nickel–phosphorus alloy during its electroless deposition and to verify the possibility of a diameter-controlled production from metal layers on the oxide surface to metal nanotubes at the bottom of the matrix. In this regard, in the presented work, the electroless nickel deposition (Ni-ELD) inside a nanoporous AAO template with average pore diameters of 13, 33, and 95 nm was investigated. The main components in the electrolyte for Ni-ELD were nickel sulphate and sodium hypophosphite, due to which nickel is co-deposited with P. The resulting Ni-P deposits were observed by scanning electron microscopy (SEM) and characterised by energy dispersive spectroscopy (EDS), both on the surface of the AAO template and in the depth of the pores. The variation in the ratio of Ni and P was investigated and discussed in relation to transport limitations in nanosized pores.

## 2. Materials and Methods

Substrates of anodised aluminium were subjected to electroless nickel plating with controllable pH 4.5. For that, technical grade aluminium foils (99%) with a thickness of 0.1 mm (Riedel-de Haen GmbH, Seelze, Germany) were degreased in acetone, then pickled in 40 g/L NaOH aqueous solution and dried. After that, the aluminium foils were anodized in three difference acids—5% phosphoric acid (H_3_PO_4_) (Merck, Darmstadt, Germany) at 120 V, 4% oxalic acid ((COOH)_2_) (Alfa Aesar, Karlsruhe, Germany) at 40 V, and 10% sulphuric acid (H_2_SO_4_) (Merck, Germany) at 20 V—to form nanoporous oxide layer with different thickness and pore diameters (Table 1).

The AAO@Al samples were used as substrates to produce conductive Ni layers by conventional electroless deposition. This technique requires preliminary surface catalysis, which is performed by sequential treatments in tin sulphate (SnSO_4_) (35 mM) (Valerus, Sofia, Bulgaria) for 3 min and in palladium sulphate (PdSO_4_) solution (Alfa Aesar, Germany) for 5 min. Electroless deposition was performed by using a conventional solution for electroless nickel containing 22 g/L of nickel sulphate heptahydrate (NiSO_4_ × 7H_2_O) (Valerus, Bulgaria) as a source of nickel ions, 20 g/L of sodium hypophosphite (NaH_2_PO_2_) (Valerus, Bulgaria) as a reducing agent, 10 g/L sodium acetate (CH_3_COONa) as a complexing agent (Valerus, Bulgaria), and 0.1 mM lead acetate (Pb(CH_3_COO)_2_) (Alfa Aesar, Germany) as a stabiliser. Electroless Ni deposition onto AAO with different designs was performed at 65 °C for 10 min. In the current work, over 5 separate samples of each type were prepared. Additionally, a copper layer was electrochemically deposited at 15 mA/cm^2^ for 10 min from an acidic copper sulphate solution onto some Ni-ELD conductive layers to check the morphology of nanocomposites (present in Supplementary Information).

Structural characterisation was carried-out by X-ray diffraction (Rigaku D/Max-B, Cu Ka) at room temperature on a sample with a layer of AAO of each type before and after electroless nickel deposition. The diffraction angle was up to 60–70° and the scanning rate was 3°/min, with a sampling step of 0.02°. The thickness and structure of the prepared nanocomposite Ni-P@AAO/Al layers and the pore structures of were characterised by scanning electron microscopy (SEM) Tescan LYRA (TESCAN, Brno, Czech Republic) equipped with an anergy-dispersive X-ray analyser (Bruker AXS GmbH, Karlsruhe, Germany). Energy-dispersive X-ray spectroscopy (EDS) in point (in a volume of about 1 μm^3^) and line scan was used for the analysis of elemental distribution in the formed layer and pores of AAO. The distribution of nickel and phosphorus elements in the depth of the Ni-P@AAO nanocomposite layers was also determined by EDS analysis, being performed at several points of the cross-section of the samples and by line scanning. Individual samples were also analysed using a field-emission scanning electron microscope JEOL IT800SHL (JEOL Ltd., Tokyo, Japan) with both secondary and backscattered electron detectors placed in chamber and in a lens microscope column. An electron gun has accelerating voltage of 5 kV. A Magnetic Properties Measurement System (MPMS 3) SQUID magnetometer (Quantum Design, San Diego, CA, USA) was used to study magnetic properties with ≤10^−8^ emu sensitivity and a field changing resolution of 0.33 Oe. Magnetization was measured at a fixed temperature of 300 K and with magnetic fields ranging from −7 to 7 T.

## 3. Results and Discussion

### 3.1. Influence of AAO Pore Size on the Morphology of Ni-ELD Coating

**Figure 1:**
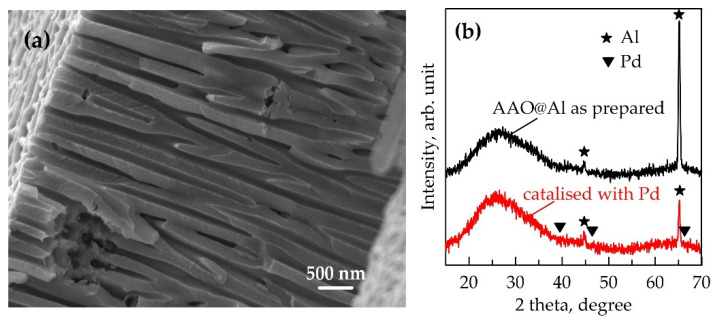
Characterisation of the anodic aluminium oxide template: (**a**) cross-section SEM image of AAO_95_ @Al structure prepared in H_3_PO_4_, ×22k magnification; (**b**) XRD of AAO_95_ template before and after deposition of Pd seeds.

The processing parameters of the anodization of pre-cleaned Al are presented in Table 1. The AAO layers with average thickness of ~10 μm were prepared in 5% H_3_PO_4_, 4% (COOH)_2_, and 10% H_2_SO_4_. The average pore diameter was calculated based on plan-view images obtained by SEM analysis as follows: ~95 ± 12 nm for AAO prepared in H_3_PO_4_ (designated here as AAO_95_), 33 ± 2 nm for AAO prepared in (COOH)_2_ (designated here as AAO_33_), and 12.5 ± 0.1 nm for AAO prepared in H_2_SO_4_ (designated here as AAO_13_), respectively. These values are consistent with those determined in our previous works [38,39,40]. A typical cross-section of AAO and its XRD profiles before and after the catalysis process are presented for AAO_95_ in Figure 1a,b, respectively. Only weak peaks at 44.6° and 65.2°, corresponding to the aluminium substrate, are observed. XRD diffractogram for the templates AAO_33_ and AAO_13_ are analogous.

**Figure 2:**
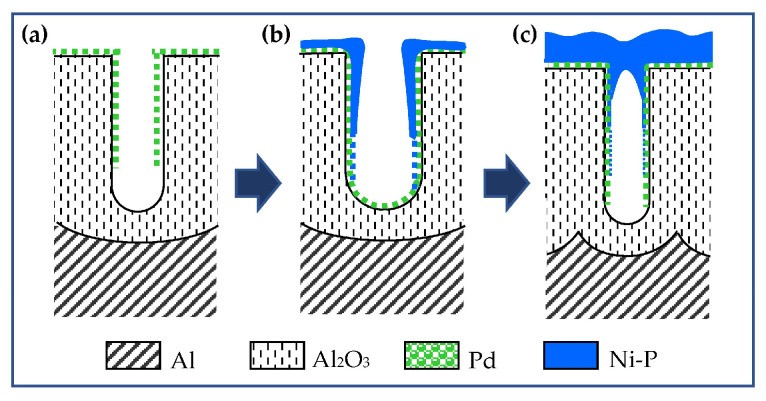
Schematic representation of the stages of AAO processing during metallization: (**a**) catalysis with Pd; (**b**) initial Ni-ELD or deposited into large pores as AAO_95_; and (**c**) Ni-ELD on small pores as AAO_33_ and AAO_13_.

The obtained templates were activated with palladium. The catalytic palladium seeds appear to be very small in size. This is probably why palladium is not detected in the diffraction patterns even for the AAO_95_, which has the widest pores and therefore the largest amount of palladium inside them (Figure 1b). However, the presence of palladium is proven by the normal course of the electroless Ni deposition process schematized in a Figure 2. Within the first minute, visually, the surface of all samples becomes deep black. This optical effect is a result of metal filing (Figure 2b) of the pores of the transparent template and light scattering by the resulting metal nanostructure. The surfaces of AAO_13_ and AAO_33_ acquire a metallic lustrous appearance after about 2 and 5 min, respectively, which indicates the formation of a compact metal layer on AAO (Figure 2c). Only AAO_95_ retains its blackness after 10 min. Similar optical surface effects in electroless nickel plating of AAO have been reported by Lin and al., who associated the black colour with the formation of a conformal Ni-P deposition [41].

**Figure 3:**
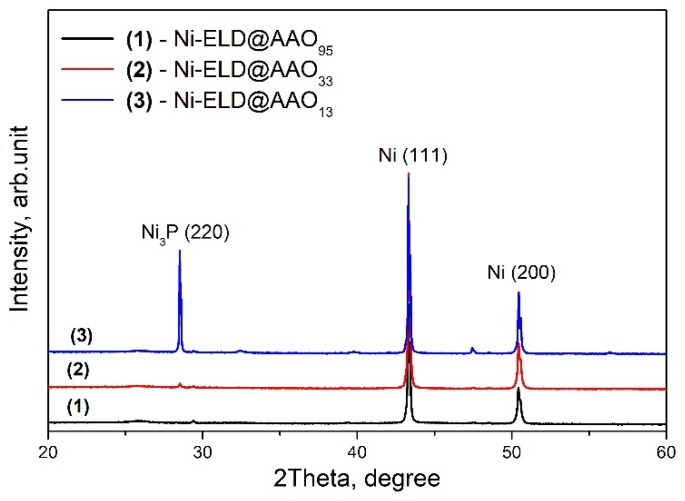
XRD profiles of electroless Ni deposited on AAO templates with pore sizes of 13, 33, and 95 nm.

Electroless Ni layers prepared on three AAO templates with different pore sizes were studied by XRD and presented in Figure 3. Strong Ni peaks were detected for all studied samples and can be associated with Ni (111) and Ni (200), according to the card # 01-077-9326. At the same time, a peak at ~28.5° was simply detected in the sample made on the template with the smallest pore size (AAO_13_) and it can be associated with Ni_3_P (220), according to the card # 01-074-1384.

The crystallite sizes (in nm) of the tested samples were calculated based on the obtained XRD profiles, in particular on Ni (111) peaks, and according to the Scherrer equation:

(1) $$D=\frac{K\times \lambda }{\beta \times \cos\Theta }$$

where *K* is the constant of Scherrer (*K* = 0.94), *λ* is the wavelength of CuKα radiation (λ = 0.15,406 nm), *β* is full width at half-maximum (FWHM), and *Θ* is peak position.

The crystallite sizes were calculated to be very similar for all samples: ~2 nm for the Ni layer onto AAO_13_ and onto AAO_95_, and ~2.4 nm for the Ni layer onto AAO_33_. These values are well correlated with the reported 5 nm crystallite size for unannealed electroless Ni layer deposited on steel substrate and that can increase with temperature [42]. Thus, all samples can be characterised by the formation of some Ni nanocrystals with a very similar size; however, only the Ni-P layer deposited onto AAO_13_ can clearly present Ni_3_P that could be due to the very small pore size of AAO and will be discussed below.

**Figure 4:**
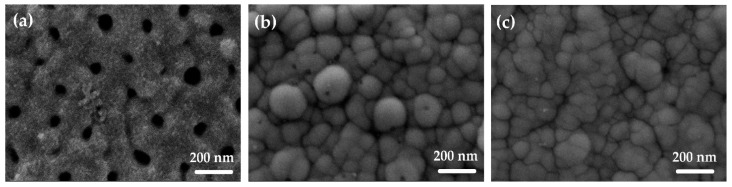
Plan-view SEM images (×100k magnification) of Ni-ELD on (**a**) AAO_95_, (**b**) AAO_33_, and (**c**) AAO_13_.

The morphology and structure of the prepared electroless Ni coating layer deposited onto AAO templates were studied by SEM and EDS. Figure 4 shows that the template coverage with electroless Ni deposited for the same time of 10 min strongly depends on the AAO pore size. Although the entire surface of the specimens is covered with nickel, the pore mouths of AAO_95_ remain open (Figure 4a). At the same time, both samples with smaller pore sizes present fully covered AAO pores with a dense Ni layer, as can be seen in Figure 4b for AAO_33_ and in Figure 4c for AAO_13_. These layers show a typical Ni-P morphology with spherical formations [13].

According to the plan-view SEM images presented in Figure 4a, the Ni-ELD layer is not continuous in the case of larger pore size (95 nm) that cannot completely cover the AAO_95_ pores as presented in schematic view of Figure 4b. At the same time, the electroless Ni layer closes all pore mouths when the diameter of AAO pores is small, corresponding to the schematic view presented in Figure 2c. Although the mouths of the largest pores in AAO_95_ remain open, the thus-metallized template allows the electrochemical thickening of the metal layer. This possibility was verified by the electrodeposition of a 2 μm thick copper layer on Ni-ELD and the final Cu@Ni-P@AAO/Al structures were investigated and presented in the Supplementary Information (Figure S1). The copper was chosen as a second metal whose penetration depth into the nanopores could be easily distinguished from nickel. Thus, EDS analysis of a cross-section of Cu@Ni-P deposited onto AAO with the highest pore size of 95 nm can detect Cu up to about 0.5 μm from the pore mouth that can support the open tube structure of Ni-ELD layer in AAO_95_ (Figure S2). At the same time, samples with AAO_33_ and AAO_13_ presented a dense structure of the Ni-ELD layer deposited onto AAO, and no Cu was detected in AAO pores.

### 3.2. Distribution of Ni and P in the Depth of the Pores

**Figure 5:**
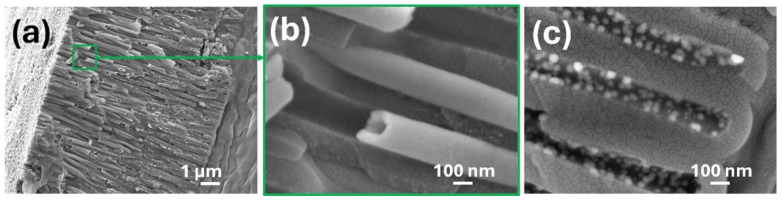
Cross-section SEM images of Ni-ELD onto AAO_95_: (**a**) composite layer overview, ×10k magnification; (**b**) enlarged part of cross-section demonstrating tube structure at small depth (near the oxide-electrolyte interface), ×100k magnification; (**c**) enlarged part of the pore bottom of AAO cross-section demonstrating individual Ni nodules (white points), ×100k magnification.

**Figure 6:**
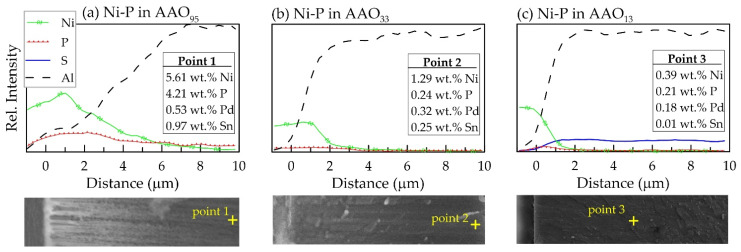
EDS analysis of element distribution (top) along cross-section (bottom images) of Ni-ELD@AAO/Al structures with 10 μm thickness: (**a**) AAO_95_, (**b**) AAO_33_, and (**c**) AAO_13_ (the legend in the upper left corner is valid for all dependencies in the figure). The symbol “+” indicates the location of analysis.

The structure of the nickel deposited along the pores was observed through a cross-section of composite layer onto AAO_95_ (Figure 5a). The cross-section SEM images of the AAO_95_ composite reveals the nanoscale diameter and up to ~5–7 μm of the length of the Ni tubes, starting from the mouth of the pores (Figure 5b). Moreover, Ni was detected along the whole length of the pores until the bottom of the AAO_95_ pores by EDS analysis (Figure 6a and Figure S2). At the same time, on the pore walls near the oxide/aluminium interface (pore bottom), numerous Ni nodules were observed (Figure 5c). Their uniform distribution suggests that the impregnation with a Pd catalyst and subsequent electroless Ni deposition were properly performed but that other factors have prevented the formation of a continuous metal film [41].

In addition to the nickel content, important information about the electroless deposition process is also obtained from that of phosphorus. This element is a residue of the sodium hypophosphite (NaH_2_PO_2_), used as a source of electrons for the reduction of nickel ions in electroless nickel deposition, which proceeds according to the following reactions:

Ni^2+^ + 2H_2_PO_2_^−^ + 2H_2_O ⟶ Ni + 2H_2_PO_3_^−^ + 2H^+^ + H_2_↑ (2)

2H_2_PO_2_^−^ + 2H^+^ ⟶ P + H_2_O + H_2_PO_3_^−^ + H_2_↑ (3)

The P content in the Ni-P coating can vary widely depending on the electrolyte composition and pH and has a strong impact on the corrosion resistance, mechanical and, magnetic properties of the layer [8]. Therefore, the industry identifies electroless nickel coatings based on P amount as follows: *low phosphorus* (<4 wt.% P); *medium phosphorus* (6–9 wt.% P); and *high phosphorus* (10–13 wt.%) [43]. According to the literature, when using hypophosphite as a reducing agent, the content of P in Ni-P deposits is in the range of 6–8 wt.% in the case of alkaline electrolytes (pH 8–10) and can reach up to 15 wt.% in acidic electrolytes (pH 4–6) [8].

In the current work, an electroless Ni layer was deposited onto different AAO from the electrolyte with pH = 4.5. EDS analysis of the plan-view of the Ni-ELD layers on AAO (Figure 4) shows that the P content varied according to the AAO substrate used. Thus, the deposits on AAO_95_, AAO_33_, and AAO_13_ contained ~10.30 ± 0.2 wt.% P, ~9.15 ± 0.4 wt.% P, and ~10.45 ± 0.3 wt.% P, respectively. In the case of the AAO_95_ template, the P contained in it must also be taken into account, which can falsely increase the registered amount of this element in the Ni-P layer. Based on the EDS study of the AAO_95_ template prepared in H_3_PO_4_ acid before electroless deposition, an additional amount of phosphorus (~1 wt.% of P) was found that it is similar to already published value of P on the walls for AAO (1.1–1.4 at.% depending on acid concentration) [44]. Anyway, the average P content among all studied samples with different pore sizes is about 9.9 wt.%, which corresponds to already published results obtained for different substrates and can belong to *medium–high-phosphorous coatings*.

**Figure 7:**
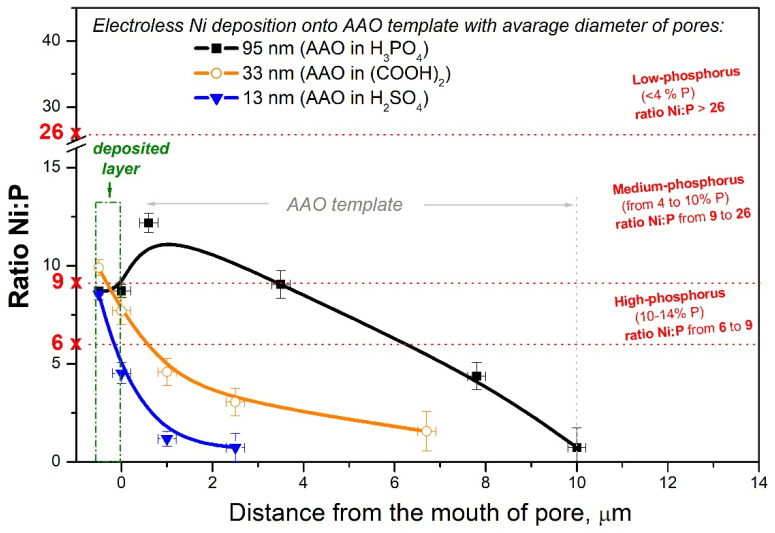
Variation in Ni/P ratio versus the distance from the mouth of the pores for AAO with diameters of pore 95 nm, 33 nm, and 13 nm.

However, it is more interesting to note that the content of P element in the Ni-P electroless deposit and consequently the Ni–P ratio changes significantly in the depth of the nanopores depending on the pore size of AAO (Figure 6, Figure 7 and Figure S2). In addition to aluminium and oxygen, the EDS-line analysis in the cross-section of Ni-ELD@AAO/Al structure reveals a presense of Ni, P, Pd, and Sn, although in some cases the amount of Pd and Sn is near the technical error of EDS (Figure 6). Traces of S are seen on the surface of Ni-ELD AAO_13_ (Figure 5c) due to incorporation of sulphate ions from sulfuric acid during anodic oxide growth.

As can be seen in Figure 6a, the dependence of Ni passes through a maximum at ~1 μm (very near to mouth of the pore) in the sample with largest pore diameter (AAO_95_). Moreover, Ni is recorded in an amount of ~5.61 wt.% at 10 μm depth, which corresponds to the full Al/AAO interface in this sample. However, the EDS analysis of an individual node at pore bottom of AAO_95_ shows a result of 21.85 ± 0.33 wt.% Ni and 3.03 ± 0.19 wt.% P, which corresponds to Ni/P = 7.2 and 12.2 wt.% P in the Ni-P layer.

In the sample on AAO_33_, with three times thinner pores of 33 nm, the Ni content sharply decreases at ~2 μm and stays at ~1 wt.% (almost covered by EDS technical error bar) up to ~8–9 μm distance from mouth of the pore (Figure 6b). A similar tendency of Ni distribution can be seen in Figure 6c, presenting data for the sample with the narrowest 13 nm pores (AAO_13_): a sharp decrease in the nickel content to about ~0.5–1 μm, and negligibly low Ni content (below 0.4 wt.% Ni and within the measurement error) at a depth of more than ~5 μm. Such distribution of Ni supports the schematic structures of the pores proposed in Figure 2; the Ni-ELD layer deposited onto AAO_95_ cannot totally cover the pore mouths (Figure 2b), in contrast to that on AAO prepared in (COOH)_2_ and H_2_SO_4_ with fully covered pores (Figure 2c).

A similar absence of the detection of Ni starting from a depth of 2 μm was reported at pore sealing by Cartigny et al. for AAO with columnar porosities of about 10 nm diameter [45] and by Rocca et al. for AAO with a pore diameter of <20 nm [46]. At the same time, Lin et al. reported a successfully deposited tubelike Ni-P structure by electroless Ni coating onto an AAO template possessing very large pores with 250 nm diameter and 10 μm length [41].

As was already mentioned above, the electroless Ni layer contains about 10 wt.% P and 90 wt.% Ni (corresponding to Ni/P ratio of 90:10 = 9) and belongs to the range of *high-phosphorus coatings* [43]. In the current work, variation in the Ni/P ratio along pores was calculated for all studied samples with different diameters as presented in Figure 7. In the case of AAO_95_ prepared in H_3_PO_4_, the amount of P released in the Ni-P layer during electroless deposition is also superimposed on that of the phosphate groups included in the template. Therefore, the highest content of P (or, respectively, the lowest Ni/P ratio) in AAO_95_ could be expected. However, an opposite trend can be observed in Figure 7 (black square for sample with AAO_95_). In general, with a decrease in the diameter of the pores as well as with an increase in depth, the Ni/P ratio becomes lower and lower (Figure 7), which indicates that the content of phosphorus relative to nickel increases inside the pores.

At a Ni/P ratio below 6, it is obviously not possible for a dense metal to form, only individual nodes (white points in Figure 5c). At a Ni/P ratio below 1, the amount of P is higher than that of Ni. The decrease in the Ni/P ratio in the depth of the pores shows that hypophosphite ions penetrate more easily and to a greater depth compared to Ni^2+^ ions. These results mean that the nanoporous AAO template “filters” the electrolyte with respect to nickel ions and allows hypophosphite ions to penetrate deep into the pores preferentially. The Ni/P ratio below 1 is reached at depths of 10, 6, and 1 μm in AAO_95_, AAO_33_, and AAO_13_, respectively.

In order to check the penetration depth of Ni^2+^ ions, electroless Ni was also deposited on two more AAO templates for comparison: AAO with the same pore dimeter (AAO_95_) but the thickness increased to ~30 μm (obtained at the same applied voltage during anodization but at longer time) and AAO with an even bigger ~125 nm pore size (AAO_125_) and with the same thickness of AAO of ~10 μm (obtained at the higher applied voltage of 140 V during anodization and similar time). SEM images of cross-sections of Ni-ELD on a 30 µm AAO_95_ template and on 10 µm AAO_125_ are presented in Figure S3 in the Supplementary Information. The quantities of the elements for Ni-ELD on the very thick AAO_95_ template are presented in Figure S3a. It is obvious that the traces of palladium and nickel are registered in AAO_95_ pores at a significant depth (Figure S3a) at aspect ratios above 300. There are no continuous nanotubes at a depth of 30 μm in this sample. At the same time, the big pore diameter in AAO_125_ supports the formation of long nanotubes (at least 10 μm in length).

The uniform deposition of metal inside the pores is determined by the transport of the main components of the electrolyte for electroless deposition which can be realised by means of diffusion and/or migration. The *diffusion* of nickel ions and the reducing agent (hypophosphite) inside the pores occurs during the initial wetting of dry samples or soaking of wet ones with the solution for electroless nickel deposition and continues throughout the process due to their depletion in the course of the chemical reaction. Thus, in this process, the concentration of both nickel ions Ni^2+^ and hypophosphite ions H_2_PO_2_^−^ at the bottom of the pores tends to zero. Moreover, the diffusion rate is determined not only by the concentration gradient, but also by the size of the ionic and hydrated shell of the reagents. Thus, the larger the size of the ion (including its ionic or hydrate shell), the slower the expected diffusion rate in aqueous solution [47]. Besides the fact that cations generally have a larger hydration shell compared to anions [48], nickel ions are additionally complexed by acetate ions in the electroless solution. The spatial limitations related to the dimensions of the ionic and hydrated shell of the ions also determines their mobility in nanopores.

It should not be forgotten that the hindered diffusion concerns not only the delivery of new reagents, but also the removal of reaction products. The diffusion mechanism in nanopores during electroless nickel deposition may become even more difficult to explain when a gaseous product is taken into account ((Equations (2) and (3)). The H_2_ released in the course of the reaction may further hinder diffusion in the pores. Additionally, in the strongly limited electrolyte volume in the pores, it could be assumed also that the hydrogen cations generated during the process (Equation (2)) accumulate in the interior of the pores, lowering the local pH, which favours an increase in the P content in the Ni-P deposit [49]. Therefore, the narrower the pores, the more difficult the diffusion in them and all the effects that are determined by it will be.

Undoubtedly, the diameter of the pores has a decisive importance for the depth of penetration of electrolyte components into their interior. However, it can be expected that a diameter above 13 nm should not be limiting for the diffusion of hydrated ion with a diameter less than 0.5 nm [47]. Because of this, traces of palladium and tin are found even at the dept of the narrowest pores (Figure 5c). For this reason, there are probably other factors that influence the differential entry of cations and anions into the pores, such as their charge. As with any solid–liquid interface, it is expected that the AAO template is charged either with a primary charge (during oxide growth) or by adsorbed ions during catalysis. As both reagents are ions (Ni^2+^ and H_2_PO_2_^−^), their *migration* under the electric field of pore walls could also be a likely reason for the different penetration depths into the pores of nickel and hypophosphite ions. Although the oxide layer is not under the influence of an external electric field as in electrodeposition, it still has its own charge. If the AAO template is positively charged before immersion in the electrolyte for electroless deposition, this would explain the easier penetration and retention of negatively charged hypophosphite ions inside the nanopores.

It is well known that AAO is characterised by an amorphous structure (see XRD in Figure 1b), with the outer layer of the pore walls incorporating anions from the electrolyte during its preparation. These anions could have an impact on the subsequent interaction of AAO with electrolytes. Unfortunately, the data in the literature on the charge of AAO are contradictory. For example, Rocca et al. supported the idea that the surface of the oxide layer inside the very narrow pores (diameter < 20 nm made in H_2_SO_4_ at 18 V) is positively charged (–OH_2_^+^ on the surface) due to the electric field fills the entire volume of the nanopores [34]. Other authors have also found that the outer boundary (AAO/electrolyte) is negatively charged, while a positive charge is recorded in the inner layers of AAO [35,36,37]. Moreover, Vrublevsky et al. demonstrated that the charge of the AAO is determined by the anodization voltage, with the limit value being 38 V; above it, the membrane is positively charged and below it, negatively [50]. That is, at 38–39 V, the AAO surface has zero charge. In an acidic electrolyte, H_3_O^+^ ions are injected into the oxide, which reduce the negative charge. The penetration depth of these injected protons increases with increasing anodization potential. However, whatever the initial charge of the AAO template, it most likely changes during subsequent treatments in a sensitising solution of SnSO_4_ and an activating solution of PdSO_4_. Judging by the fact that anions penetrate more easily into the pore depth, it can be indirectly assumed that after catalysis the charge of the pore walls is positive. These conclusions are also based on the studies of Rocca et al., according to which in narrow pores (a diameter of <20 nm) with positively charged walls, the diffusion of negative particles inside the AAO nanopores is faster than the diffusion of cationic particles [46]. Thus, in the case of small nanometre pores, the electric field (which extends for about 10 nm in a dilute electrolyte [51]) created by the charged surfaces can completely inhibit the diffusion of same charged ions. For larger pore diameters (>100 nm), such an effect is not observed [52]. Indeed, in our work, nickel species enter to the bottom of AAO_95_ at a depth of over 10 μm. Moreover, when the pore diameter is increased to an average of 125 nm, a conformal Ni-P coating is deposited along the entire length of the pores until nanotubes are formed (Figure S3).

Thus, based on the information above, the kinetics of metal particle incorporation can be tuned by the diameter and the surface charge density inside the nanoscale pores of AAO, namely, the type of acid used, the magnitude of the voltage, and the electrolyte components.

### 3.3. Magnetic Test Results

The study of the magnetic properties of Ni-P layers deposited on AAO templates seeks to answer two main questions. The first question is whether nanotubes can influence the orientation of the magnetic field. In practice, the Ni-P layer has a 3D configuration, consisting of a flat “plate” and nanotubes arranged perpendicular to it. The second important question is how changing the Ni/P ratio in metal nanotube affects the magnetic properties of this 3D configuration. While nickel is known to be a ferromagnetic material, Ni-P deposits with P contents greater than 11% are described as essentially non-magnetic [43].

**Figure 8:**
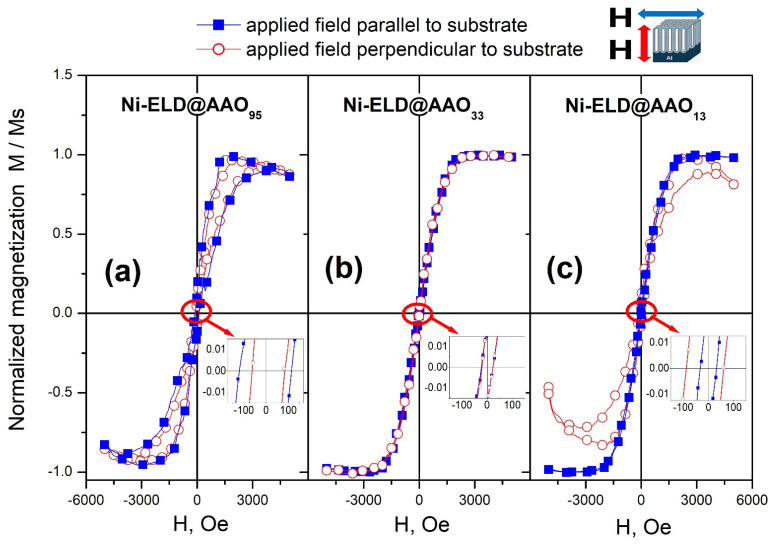
Normalised magnetization of the electroless Ni-P deposited on AAO_95_ (**a**), AAO_33_ (**b**), and AAO_13_ (**c**). Inserts are enlarged M/Ms vs. H for each sample. The magnetic field was applied parallel to substrate direction (blue closed square) and perpendicular to substrate (red open circle) during the measurements. Data were collected at room temperature.

The normalised magnetization curves (M/Ms vs. H) for Ni-ELD deposited on different AAO are presented in Figure 8. As can be seen, Ni deposited onto AAO_95_ (made in H_3_PO_4_) (Figure 8a) and onto AAO_33_ (made in oxalic acid) (Figure 8b) have similar curves in both measured directions: along and perpendicular to pores. In contrast, Ni-ELD deposited onto AAO_13_ with the smallest pore diameter shows slightly different magnetic responses in parallel and perpendicular directions (Figure 8c), which can be explained by the presence of a higher amount of material with vertically oriented magnetic moments only in the top surface layer but not inside the AAO pores (no Ni in AAO_13_ pore depth was confirmed by SEM and EDS).

It should be emphasised that due to the fact that not only the AAO pores can contain electroless deposited magnetic materials (tubes/nanorods are formed), but also the surfaces of the samples (Ni-P film), the explanation of the measurement results is likely related to the different places of the concentration of magnetic Ni in formed Ni-P coatings:

- Ni can be concentrated mainly in 5–7 μm tubes inside AAO_95_, as was supported by EDS;
- Ni can be presented in both Ni-P film on the top of AAO as well as in the formed very short nanotubes/nanorods in AAO_33_;
- Ni can be concentrated only in the top film layer in the case of using the AAO_13_.

At the same time, the coercive field H_c_ decreases from ~120 Oe for EL-Ni deposited on AAO_95_ (Figure 8a, inset) to ~25–30 Oe for EL-Ni deposited on AAO_33_ (Figure 8b, inset) and on AAO_13_ (Figure 8c, inset). McHenry et al. reported that such low values of H_c_ were found for materials with a nanocrystalline structure [53]. It was explained by the total disorder of magnetic moments in amorphous/nanocrystalline structures.

Similar very thin hysteresis loops were observed by Huang et al. for Ni arrays prepared by electroless on AAO with ~70 nm pores on a Si substrate [54]. Huang et al. mentioned the paramagnetic state for the obtained Ni array and the highest values of the magnetization were found to be less than 2 × 10^−4^ emu at 10,000 Oe. However, the ferromagnetic ordering was gradually restored after post-annealing. This was explained by the high level of crystallinity after heat treatment that results in the formation and ordering of ferromagnetic domains that were not observed in the as-deposited Ni array. The short-range ordering was reported by Huang et al. for the as-deposited electroless Ni arrays, and the crystallite has diameter of 2–3 nm, similar to the ~2–2.4 nm crystalline size calculated for the currently studied samples based on XRD profiles, presented in Figure 2. Ren et al. compared Ni nanowires and nanotubes electroless deposited on AAO with pores of 70 nm in diameter and 60 μm in length and reported on elevated H_c_ obtained in parallel to wires/tubes direction for both cases [55]. At the same time, the H_c_ for tubes were twice lower in comparison to wires. Moreover, the easily magnetised direction was reported to be parallel to the nanowire/nanotube arrays and that both the Ni–P nanowire and nanotube arrays have obvious magnetic anisotropy. The aspect ratio that was calculated to be beyond 800 was proposed as a possible explanation of such easy ordering [55]. Moreover, an easy magnetization axis that can be gradually orientated with the aspect ratio growth, and magnetization of the arrays that can be changed mainly due to shape anisotropy effect but not due to the magnetocrystalline anisotropy effect were reported by Huajun et al. [56]. However, among the samples studied here the clear and stable nanotubes were detected only for Ni-ELD on AAO_95_ and the aspect ratio for them can be calculated as ~5 μm/95 nm ≈ 53 for this sample.

Thus, it can be additionally concluded that there is very small anisotropy in the samples with 95 nm and 33 nm pore size of AAO used. This can mean that the magnetic signal likely comes from small independent particles. The Ni-ELD coating on AAO with a 13 nm pore size shows lower magnetic signal along the pores direction that could be related to the concentration of magnetic material on top of AAO but not in AAO pores.

## 4. Conclusions

Anodized aluminium oxides with different pore size were obtained by tuning acids and voltage. The palladium activation onto AAO was used for catalysis and allows for successful electroless deposition of Ni metal reduced with NaH_2_PO_2_ to Ni-P coating.

The following have been established:

(1) Within 10 min of electroless deposition, the nanopores with a diameter of ~95 nm are conformally covered and remain open, while a compact Ni-P layer is formed on the templates with nanopore diameters of ~13 and ~33 nm. The average phosphorus content in the surface deposit on all AAO templates with different pore sizes is about 9.9 wt.%, which corresponds to medium–high phosphorous coatings.
(2) At a diameter of about 95 nm, traces of nickel, phosphorus, and palladium are recorded at a depth of 30 μm at an aspect ratio of 300, but dense coverage of the pore walls observed to a depth of about 4–5 μm. As the pore diameter decreases, the Ni deposition occurs at a reduced depth and at a diameter of about 13 nm; electroless deposition occurs primarily on the surface of the oxide template (below 1 μm).
(3) The composition of the Ni-P deposit changes along the pore depth and the nickel content gradually decreases along the pores of AAO such that the Ni/P ratio decreasing to about 1 at depths of 10 μm, 6 μm, and 2 μm at pore diameters of 95, 33, and 13 nm, respectively.
(4) The different penetration depths of the Ni-P deposit are mainly attributed to spatial limitations in the diffusion processes in nanopores of both the reactants nickel and hypophosphite ions, as well as the products of the reaction and acidification of the electrolyte inside the pores. However, changing the deposit composition (the Ni/P ratio) suggests a more complex mechanism of action of the pore diameter and the charge of the oxide walls.
(5) The magnetic properties of the resulting 3D configuration of the Ni-P deposit (flat plate and nanotubes arranged perpendicular to it) are determined primarily by the penetration depth of the Ni-P deposition process (the length of the nanotubes) rather than by its composition.
(6) By varying the pore diameter of the nanostructured oxide layer, it is possible to control the depth of Ni penetration and hence obtain a sufficiently well-defined structure of the Ni-P@AAO/Al.

In conclusion, in the electroless nickel plating technology with Pd-activation of the AAO template, the depth of metal penetration and the composition of the deposit depend on the pore diameters. The technology is appropriate for obtaining nanotubes by conformal metal coating of the nanoporous template as well as dense metal surface layer by tuning the design of AAO and processing parameters. The Ni-P@AAO nanocomposite can be used as prepared or after the removal of the AAO template or after further processing for energy conversion, supercapacitors, or sensor systems.

## Figures and Tables

**Table 1:** Parameters of Al anodization process as well as the average pore diameter of the obtained AAO templates.

Name of Template	Used Acid	Applied Voltage, V	Temperature of Anodization, °C	AAO Pore Diameter, nm
AAO_95_	5% H_3_PO_4_	120	14 ± 1	~95
AAO_33_	4% (COOH)_2_	40	14 ± 1	~33
AAO_13_	10% H_2_SO_4_	20	6 ± 1	~13

## Data Availability

The original contributions presented in this study are included in the article/Supplementary Material. Further inquiries can be directed to the corresponding authors.

## Supplementary Materials

The following supplementary information can be downloaded at: https://www.mdpi.com/article/10.3390/ma18163797/s1, Figure S1: Schematic view of the electrochemically deposited copper (Cu-ED) layer onto electroless deposited nickel (Ni-ELD); Figure S2: SEM images of cross-section of AAO_95_ with electroless deposited Ni and electrochemically deposited Cu: general view (a) and enlarger middle part of the AAO/Ni-ELD interface (b). EDS analyse of Ni-ELD/Cu-ED interfaces onto AAO_95_ (c), AAO_33_ (d) and AAO_13_ (e); Figure S3: Cross-section SEM images of Ni-ELD on: 30 µm AAO_95_ template (in backscattered electron mode) (a), and in 10 µm AAO_125_ (in secondary electron mode) (b).
