# Supplementary material for: Effect of Anodic Aluminium Oxide Structure on the Electroless Ni-P Distribution into Nanopores
Source: Materials (Basel). 2025 Aug 13;18(16):3797. doi: 10.3390/ma18163797 (PMC12387956; doi:10.3390/ma18163797)
Supplement: Supplementary file 1 [file materials-18-03797-s001.zip › materials-3790208-supplementary.pdf]

Supplementary Information

Effect of anodic aluminium oxide structure on the electroless Ni-P distribution into nanopores

Boriana Tzaneva[1*], Olena Okhay[1,2*], Vesselina Milusheva[1], Stela Atanasova-Vladimirova[3], João Ventura[4], Alexander Tkach[5]

[1] Department of Chemistry, Technical University of Sofia, 1000 Sofia, Bulgaria
[2] TEMA-Centre for Mechanical Technology and Automation, Department of Mechanical Engineering, University of Aveiro, 3810-193 Aveiro, Portugal
[3] Institute of Physical Chemistry "Rostislav Kaishev", Bulgarian Academy of Sciences, 1113 Sofia, Bulgaria
[4] IFIMUP- Institute of Physics for Advanced Materials, Nanotechnology and Photonics, Departamento de Fisica e Astronomia, Faculdade de Ciências, Universidade do Porto, Rua do Campo Alegre s/n, 4169– 007 Porto, Portugal
[5] CICECO–Aveiro Institute of Materials, Department of Materials and Ceramic Engineering, University of Aveiro, 3810-193 Aveiro, Portugal

*Correspondence: borianatz@tu-sofia.bg (B.T.); olena@ua.pt (O.O.)

A copper layer (Cu-ED) was electrochemically deposited from an acidic copper sulphate solution onto Al/AAO/Ni-ELD structure to check the morphology of Ni-ELD layer (Fig. S1).

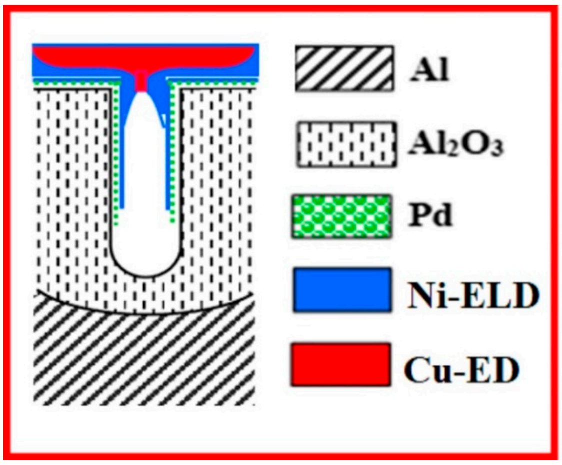

Figure S1. Schematic view of the electrochemically deposited copper (Cu-ED) layer onto electroless deposited nickel (Ni-ELD).

Cross-section SEM images of Ni-ELD with Cu-ED onto AAO with the highest pore size of 95 nm is depicted in Figure S2a and 2b. At higher magnification the presented structure of the AAO/Ni-ELD interlayer appears homogeneous in the middle part of the AAO (Fig. S2b). Well-formed Ni-ELD coatings are visible on the AAO pore walls up to about 3-5 µm from the pore mouth (based on SEM images and EDS). However, with increasing depth of the pores, the nickel layers on the walls become “island-like” and only isolated nickel deposits are visible

above 5-7 μm. Copper was detected by EDS on the top surface of the prepared sample with AAO_{95} and the weight values of Cu together with Ni, P, Al and O in the interface AAO/EL-Ni/Cu-ED are presented in Figure S2c. Cu content (~20 wt.%) was found to strongly decrease with increase of the distance from the mouth of AAO pores and was almost undetectable starting from 1 μm distance. Thus, copper was concentrated on the top of surface of the electroless deposited Ni layer (open mouth of pores) and did not enter deeply into AAO/El-Ni. Cross-sections of sample with AAO_{33} and sample with AAO_{13} were also studied by SEM, however copper was not detected by EDS analyse in these samples (Figs. S2d and S2e) that can support dense structure of deposited Ni layer onto AAO presented before on the plan-view images in Figures 4b and 4c in the manuscript.

Furthermore, the detection of Cu up to 1 μm (Fig. S2c) indicates the formation of a top-open-pore structure – nanotubes – in the case of sample with large pore diameter (95 nm) and vice versa - the total absence of Cu (Figs. S2d and S2e) for samples with small pores (13-33 nm) indicates fully covered pores in AAO with small pore diameters.

Thus, EDS study of the penetration of Cu in AAO/Ni-ELD structure resulted in fully dense Ni layer in the case of diameter of AAO pores of 33 nm (AAO_{33}) (Fig. S2d) and 13 nm (AAO_{13}) (Fig. S2e). Moreover, EDS analysis also presented that Ni distribution/amount inside the pores depends on diameter of AAO pores, in relation with other chemical elements, especially with phosphorus.

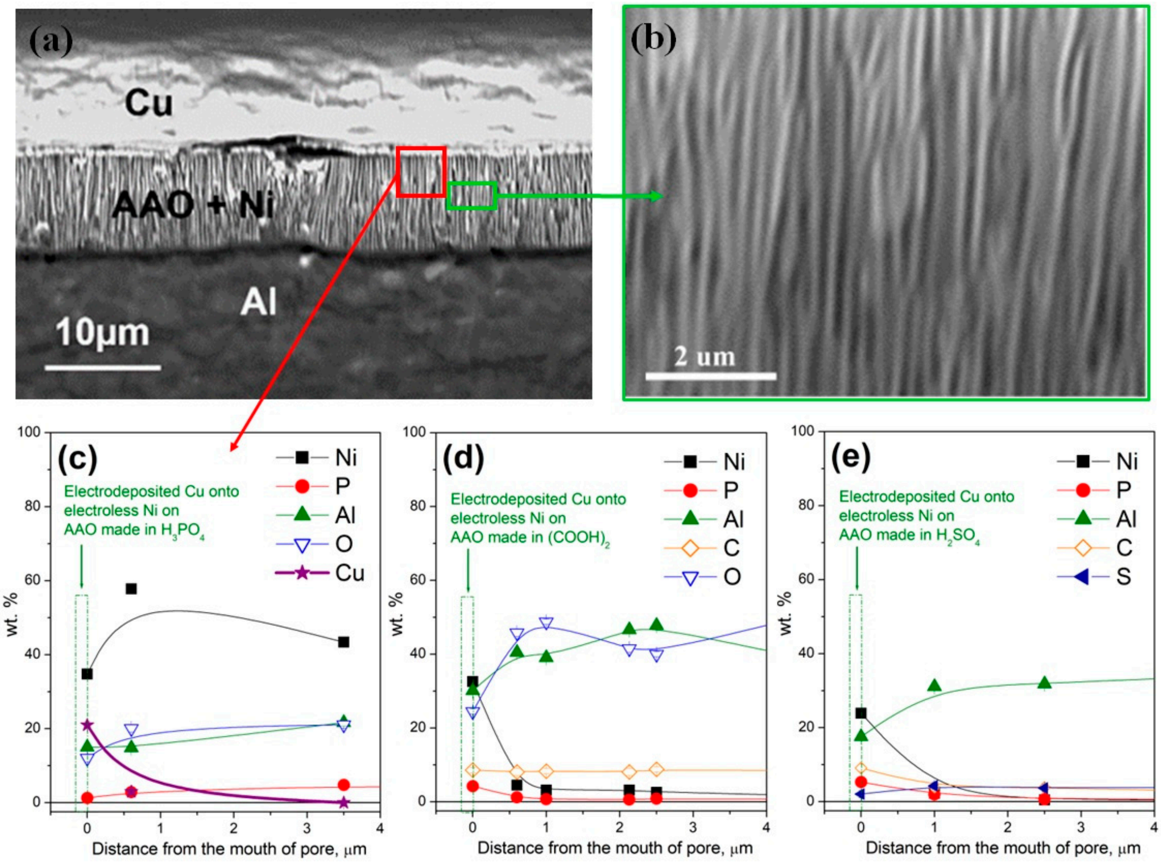

Figure S2. SEM images of cross-section of AAO_{95} with electroless deposited Ni and electrochemically deposited Cu: general view (a) and enlarger middle part of the AAO/Ni-ELD interface (b). EDS analyse of Ni-ELD/Cu-ED interfaces onto AAO_{95} (c), AAO_{33} (d) and AAO_{13} (e).

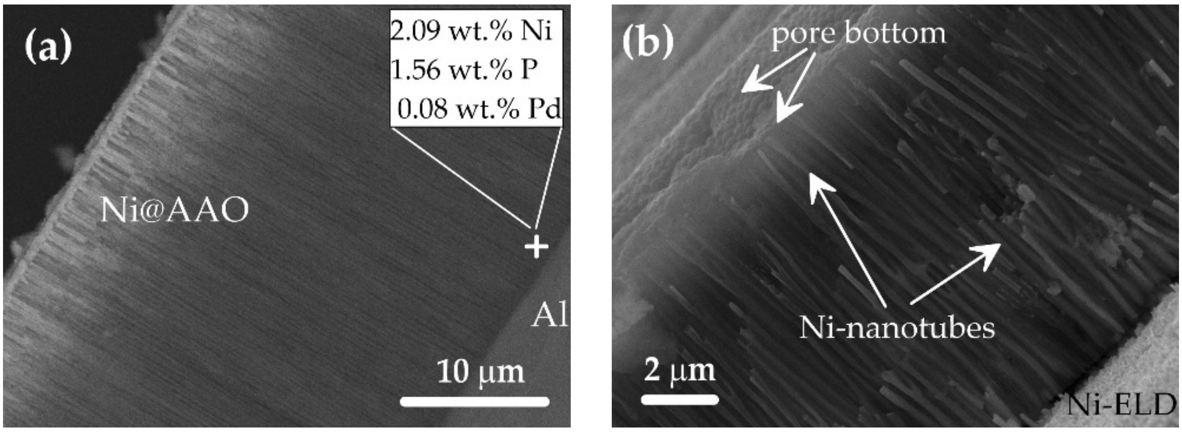

Figure S3. Cross-section SEM images of Ni-ELD on: 30 µm AAO_{95} template (in backscattered electron mode) (a), and in 10 µm AAO_{125} (in secondary electron mode) (b).
